# Supplementary material for: Association between oral microbiome and seven types of cancers in East Asian population: a two-sample Mendelian randomization analysis
Source: Front Mol Biosci. 2023 Nov 21;10:1327893. doi: 10.3389/fmolb.2023.1327893 (PMC10702768; doi:10.3389/fmolb.2023.1327893)
Supplement: Supplementary file 1 [file DataSheet1.ZIP › Supplementary Materials/MR plots for tongue/tongue═╝/Pancreatic cancer/╥╚╧┘░⌐_╔╕╤í╜ß╣√/pheno.1925_to_pancreatic cancer_forest.pdf]

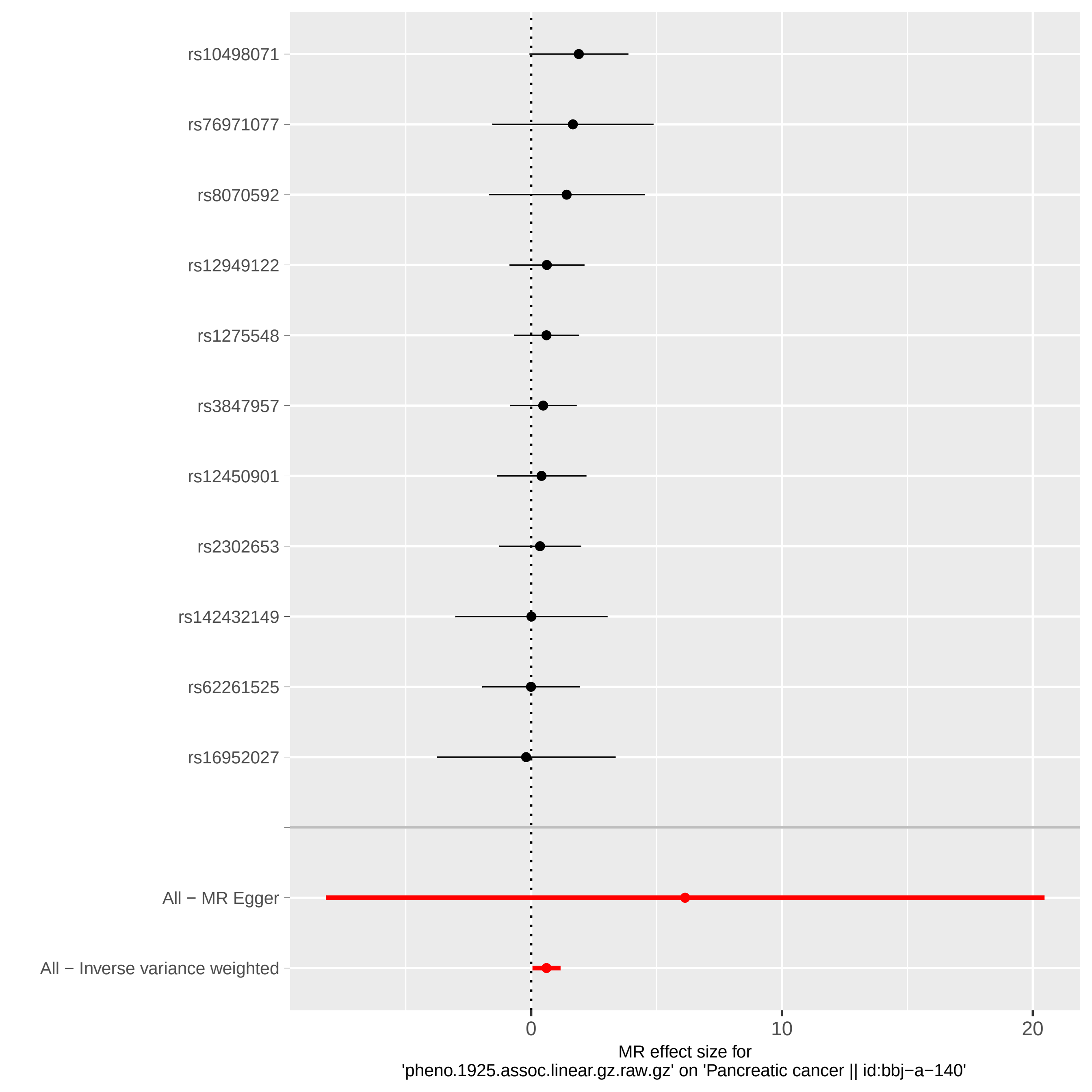
rs10498071
rs76971077
rs8070592
rs12949122
rs1275548
rs3847957
rs12450901
rs2302653
rs142432149
rs62261525
rs16952027
All − MR Egger
All − Inverse variance weighted
0
10
20
MR effect size for
'pheno.1925.assoc.linear.gz.raw.gz' on 'Pancreatic cancer || id:bbj−a−140'
